# Physical Activity Level, Insomnia and Related Impact in Medical Students in Poland

**DOI:** 10.3390/ijerph18063081

**Published:** 2021-03-17

**Authors:** Magdalena Dąbrowska-Galas, Kuba Ptaszkowski, Jolanta Dąbrowska

**Affiliations:** 1 Department of Kinesitherapy and Special Methods, School of Health Sciences in Katowice, Medical University of Silesia, 40-752 Katowice, Poland; 2 Department of Clinical Biomechanics and Physiotherapy in Motor System Disorders, Faculty of Health Science, Wroclaw Medical University, 50-355 Wroclaw, Poland; kptaszkowski@gmail.com; 3 School of Health Sciences in Katowice, Medical University of Silesia, 40-752 Katowice, Poland; 1dabrowskajolanta@gmail.com

**Keywords:** physical activity, sleep, insomnia

## Abstract

Background: Specific academic environment and time spent on learning may lead to sleep deprivation and a sedentary lifestyle. Insomnia is the most common sleep complaint. The purposes of this study were to describe the prevalence of insomnia in medical students, and to examine physical activity levels and other behavioral factors associated with insomnia in this population group. Methods: We included 308 medical students from Poland. The International Physical Activity Questionnaire (IPAQ) was used to assess physical activity levels and the Athens Insomnia Scale (AIS) was used to assess insomnia among students. A multifactor model of analysis was used to analyze variables related to insomnia. Results: A share of 19.2% of medical students were inactive. Insomnia was reported by 36.8% of students. In the multifactorial model, variables such as smoking cigarettes (ß = 0.21, *p* < 0.001), consuming energy drinks several times a month (ß = 0.21, *p* = 0.024), or daily stress (ß = 0.44, *p* < 0.001) had a negative impact on the quality of sleep of medical students. Conclusions: Most medical students are physically active, however, approximately one-third of the medical students experience insomnia. This sleep problem is reported more often in students who experience daily stress or smoke cigarettes.

## 1. Introduction

Insomnia is the most recognized sleep disturbance and the most common sleep complaint [1,2]. Individuals suffering from insomnia have either difficulty in initiating the sleep process or maintaining sleep for more than seven hours [3]. It is reported that approximately 30% of adults suffer from insomnia [3]. Specific academic environments, increased responsibility, difficult examinations, and time spent on learning may lead to sleep deprivation [4]. Students are advised to sleep about 7 to 9 h per night [5,6]. However, medical students are particularly prone to sleep-related problems. Sleep is not considered a top priority and students tend to reduce their sleep to have extra time for studies [4,7]. Based on a large study, Gaultney et al. suggests that 40% to 77% of students suffer from poor sleep quality, reporting frequent awakening, difficulties in initiating sleep, unrestful sleep, or insufficient sleep [8]. Based on studies from various countries, the prevalence of insomnia in college students ranges from 9.4% to 56.7% [8,9,10].

About 60% of medical students experience poor sleep quality, which in turn can lead to long term insomnia [11,12], and approximately 70% of students reported insufficient sleep [13,14].

Sleep deprivation has a negative impact on physical and mental health, affecting the quality of life [2]. It may result in decreased work effectiveness, poor academic performance, psychiatric disorders, impaired immune function, and stress in students [15,16,17,18]. Researchers have shown that insomnia was related to poor memory, irritability, fatigue, concentration difficulties, headache, and poor quality of life [10,19,20].

Sleep and physical activity (PA) play a vital role in maintaining good health and cognitive functions [21,22]. However, one study showed a drastic reduction of leisure-time PA. The number of adults who reported no leisure-time PA increased from 19.1% in 1988 to 51.7% in 2010 in women and from 11.4% to 43.5% in men during the same time [23]. It has been shown that the PA level is suboptimal in most students and in medical students in particular [24,25]. In a global survey of 23 different countries, between 21.9% and 80.6% of students were physically inactive [26]. Recent studies have showed that approximately 40% of medical students had low PA levels [27]. According to the World Health Organization, it is recommended that adults aged 18–64 years should perform at least 150–300 min of moderate-intensity aerobic PA or at least 75–150 min of vigorous-intensity aerobic PA, or an equivalent combination of moderate- and vigorous-intensity activity throughout the week. Adults may increase moderate-intensity aerobic PA to more than 300 min or perform more than 150 min of vigorous-intensity aerobic PA or an equivalent combination of moderate- and vigorous-intensity activity throughout the week. Adults should also perform muscle-strengthening activities at moderate or greater intensity that involve all major muscle groups on two or more days a week, because these provide additional health benefits [28]. To the best of our knowledge, no studies have been conducted to analyze insomnia and PA levels in medical students in Poland. Therefore, the purposes of this study were to describe the prevalence of insomnia in medical students, and to examine PA levels and other behavioral factors associated with insomnia in this population group.

## 2. Materials and Methods

### 2.1. Participants

The study was conducted from February to April of 2019 in Katowice, Poland. This study used a cross-sectional design and a convenience sample of 340 students from the Medical University of Silesia in Katowice. Inclusion criteria were female or male, aged 18–35 years old. Students who refused to take part in the study or had a contraindication for PA were excluded from the study. Thirty-two subjects with missing data were excluded from the analysis, leaving 308 students included in the study.

The questionnaire was distributed by the researcher. This included students in classrooms during the rest period between classes, at open areas on campus, and in the food court. The participants were given instructions on how to complete the questionnaire and informed of the study objectives. All students signed a consent that their participation was voluntary and anonymous. The study protocol was reviewed and approved by the Bioethical Committee of the Medical University of Silesia in Katowice (KNW/0022/KB/103/18).

### 2.2. Outcome Measure

The survey consisted of 3 parts: the first part included questions regarding the socio-demographics and lifestyle characteristics; the second part was the Athens Insomnia Scale (AIS); and the third part included the International Physical Activity Questionnaire-Short Form (IPAQ-SF). The sociodemographic research included questions such as age, gender, weight, height, economic status, place of residence, tobacco use, coffee consumption, consumption of energy drinks (several times a month/several times a week/I do not drink), and alcohol consumption (occasionally/once a week/every day/I do not drink). Stress was also analyzed as an independent variable in the current study. Students were asked: “How often have you experienced stress during the last 2 months?”. Students could choose one answer: “never/very rarely/several times a month/several times a week/every day”.

The AIS is an eight-item self-reported questionnaire that measures the intensity and effect of insomnia based in the International Statistical Classification of Disease and Related Health Problems - 10th Revision (ICD-10) diagnostic criteria for insomnia [29]. The first 5 items assess difficulty with sleep induction, awakening during the night, early morning awakening, total sleep time, and overall quality of sleep. The remaining 3 items pertain to the sense of well-being, overall functioning, and sleepiness during the day. Respondents were required to rate positively if they experienced sleep difficulties at least thrice per week during the last month. A total score was obtained after summing all responses, and the total score ranged between 0 and 24, where the commonly accepted cut-off is 6. Additionally, a higher AIS score indicates a higher level of insomnia. Each item is rated on a 4-point numerical rating scale (where 0 = “no problem at all” and 3 = “very serious problem”). This questionnaire is a scale used in a large variety of clinical and research settings where the quantification of insomnia is required. The AIS has remarkable psychometric properties with a Cronbach’s alpha of 0.9 [30,31].

IPAQ-SF is a self-reported questionnaire evaluating the PA level among adults ranging from 15 to 69 years of age [32]. This measurement has shown reliability and validity within different contexts, including the Polish population [33]. The IPAQ-SF consists of 7 items and records PA at vigorous intensity (hard effort, causing an increased breathing rate and heartbeat, e.g., running, aerobics, sport cycling, tennis), moderate-intensity activities (average effort with a slightly increased breathing rate and heartbeat, e.g., swimming, yoga, or recreational cycling) and walking (marching, Nordic walking, walking). The IPAQ-SF also asks participants about time spent sitting. The IPAQ-SF asks about activities lasting longer than 10 min in the 7 days prior to completing the questionnaire. PA level was expressed as Metabolic Equivalent of Work (MET)-min per week and calculated by multiplying the MET assigned to it (vigorous—8 MET, moderate—4 MET, and walking—3.3 MET) by the number of days it was performed during the previous 7 days [32,34]. The total PA of the participants was divided into 3 categories: high, moderate, and low.

A high PA level included any one of the following two criteria:

- vigorous activity on at least three days resulting in at least 1500 MET-minutes/week (MET-Metabolic Equivalent of Work) or;
- 7 or more days per week with any combination of walking, moderate-intensity or vigorous activities representing at least 3000 MET-minutes/week.

A moderate PA level included any one of the following three criteria:

- 3 or more days per week with vigorous activity for at least 20 min per day or;
- 5 or more days per week with moderately intense activity or walking for at least 30 min per day or;
- 5 or more days per week with any combination of walking, moderate-intensity, or vigorous intensity activities achieving a minimum of at least 600 MET-min/week.

A low PA level included:

Those individuals who did not meet criteria for categories “high” or “moderate” were considered low/inactive [32].

### 2.3. Statistical Analysis

Statistical analysis was performed using the Statistica 13 program (TIBCO Inc.; Palo Alto, CA, USA). The frequency of reporting sleep problems in physically active and inactive people was compared. The fraction in one group was 0.16, and in the other, 0.26. The alpha level was set at 0.05, and the power of the test at 0.8. On the basis of the parameters, the estimated sample size was equal to 257 people. In addition, due to the possibility of incomplete results of the questionnaires by the participants, the final group was assumed to increase by 20%. The final sample size equaled 308 participants. Arithmetic means, standard deviations, and range of variability (extreme values) were calculated for measurable variables. Frequency of occurrence (percent) was calculated for qualitative variables. All quantitative type variables were checked using the Shapiro–Wilk test to determine the type of distribution. Differences in gender according to PA level were analyzed using the chi-square test. The level of α = 0.05 was used for all comparisons.

In addition, an analysis of the impact of selected factors on AIS was performed using a linear regression (one-factor model of predictors included in the analysis). A non-standardized and standardized regression coefficient, standard error, and level of statistical significance were determined. The next step was the construction of the multifactor model (progressive step method), taking into account the variables whose *p*-value in the univariate model was less than or equal to 0.05.

## 3. Results

The study sample (*n* = 308) was approximately half women (56.8%), with good economic status (60.1%). Study participants were 20.0 ± 1.2 (mean ± SE) years old, and all students consumed coffee, 29.6% of students experienced stress every day, and 19.2% of participants were physically inactive. According to AIS, insomnia was reported in 36.8% of medical students. The mean value of total PA level was 1839 (SD 1161) MET-min/week (Table 1).

Differences in PA level between males and females were observed (Table 2). Significantly more males (64.38%) than females (35.62%) achieved vigorous PA level. Females were significant more likely to present a sedentary lifestyle (77.97%).

The correlation analysis of the results of the AIS and IPAQ questionnaires is presented in Table 3. There is no statistically significant correlation between the evaluated variables (Table 3).

The assessment of the impact of selected parameters on AIS (one-factor model of predictors included in the analysis) is presented in Table 4. A non-standardized and standardized regression coefficient, standard error, and the level of statistical significance were determined. The following variables were included in the analysis: age, BMI, IPAQ questionnaire results, gender, economic status, place of residence, tobacco use, stressful situations, and consumption of energy drinks and alcohol.

Linear regression analysis in a single-factor model showed the impact of gender (women), poor economic status, cigarette smoking, daily stressful situations, and subjectively assessed PA on deterioration of insomnia. Students who subjectively perceive themselves as physically active, with a higher age, positive economic status, and experiencing stressful situations several times a month, were less likely to report insomnia symptoms. In the multifactorial model, variables such as smoking cigarettes, consuming energy drinks several times a month, or experiencing daily stressful situations confirmed the deterioration of insomnia.

## 4. Discussion

Insomnia is one of the most common sleep disorders and is increasing globally [1]. There are many studies assessing the prevalence of insomnia among students throughout the world.

A systematic review on the prevalence of insomnia among students showed that the prevalence of insomnia ranged from 9.4% to 56.7% [8,9,10]. Another study conducted in Hong Kong showed that the prevalence of insomnia among students was 68.8% [35].

Our findings revealed that insomnia was reported by approximately 37% of medical students. These findings are consistent with a study carried out by Alsaggaf M.A et al., where 320 medical students were investigated in a cross-sectional study and the prevalence of insomnia was 33% [7]. The prevalence of insomnia in our results was higher than the prevalence in Lebanon (10.6%) [36] and considerably lower than in Germany (51.9%) [37], Hong Kong (68.8%) [35], Ethiopia (61.6%) [38], and Turkey (75.9%) [39].

Our findings revealed that, according to IPAQ, approximately 19% of medical students were not physically active and females were significantly more likely to present a sedentary lifestyle. Almost 81% met PA guidelines. This is consistent with a study of Stanford F.C. et al. who reported that 84% of medical students met PA guidelines [40]. This is also in line with other studies that showed that medical students engaged in more PA than the general population [41,42].

However, different studies have shown that approximately 40% of medical students had low PA levels [43]. In this study, the instruments used to evaluate PA level varied. In addition, however, factors such as schedule at the university or availability of sports centers could be considered. PA has a wide variety of health benefits, furthermore, healthcare providers are expected to recommend healthy habits to their patients. Evidence shows that active doctors prescribe PA to their patients, whereas inactive doctors are less likely to provide exercise counseling [44,45]. There are several studies that reported a relationship between PA and sleep quality [29,42,44,45,46]. However, correlations have not previously been assessed between insomnia and PA in students. In our findings, interestingly, multifactorial regression analysis revealed no significant association between PA level, assessed according to IPAQ, and insomnia. Despite the absence of a significant difference between PA levels and insomnia symptoms, our results showed that students who subjectively perceived themselves as physically active were less likely to report insomnia symptoms.

We revealed that insomnia is associated with gender (women), poor economic status, daily stress, cigarette smoking, and subjectively assessed PA. After multifactorial analysis, we found that factors such as experiencing stressful situations daily, smoking cigarettes, and consuming energy drinks several times a month were independent risk factors for insomnia in medical students.

A high prevalence of stress in medical students is commonly reported in many studies [3,4,8,21,38]

Our results showed that everyday stress was independently associated with insomnia in medical students. These findings are supported by other studies, which showed a significant association between higher levels of stress and insomnia in medical students [8,46,47,48]. The possible cause of the association between insomnia and stress, in addition to academic factors, may be poor economic relationships. We demonstrated that this factor increased the risk of insomnia in students and other studies showed similar results [48,49].

The literature shows that students at medical and sports universities are more likely to consume energy drinks to obtain better grades [50,51]. The main component of energy drinks is caffeine, and many studies found a strong association between caffeine and sleep complaints [14,52,53].

Our findings revealed that students who reported energy drink consumption several times a month experienced insomnia more often. There was no significant correlation between insomnia and energy drink consumption a few times a week. The reason for this could be a developed dependence on caffeine in students who consume energy drinks more often.

Consistent with previous studies [54,55], our results showed a significant association between smoking and insomnia. Students who smoked cigarettes were more likely to suffer from insomnia.

However, energy drink consumption and smoking were not sufficiently assessed in the current study. Therefore, further studies are needed to assess the relationship between insomnia, type and consumption time of caffeinated beverages, and number of cigarettes and length of time of smokers.

### Limitations of the Study

First, results need to be interpreted with caution because the study sample represents a convenience sample of students from a single medical university. Therefore, results may not be generalized to students at other universities. Second, factors such as gender (women), poor economic status, cigarette smoking, daily stressful situations, and subjectively assessed PA influenced insomnia, however many other factors and sleep disorders, potentially confounding the diagnosis of insomnia, were not evaluated. Third, stress was assessed by asking students about the frequency of stress experienced, which may be biased compared to studies conducted utilizing diagnostic interview tools. Further studies should employ a longitudinal research design to better explore the casual relationship among insomnia and other important variables.

## 5. Conclusions

Approximately one-third of medical students experience insomnia. This sleep problem is reported more often in students who experience daily stress or tobacco consumption. In addition, medical students who report energy drink consumption several times a month experience insomnia more often. Most medical students are physically active, however, PA level did not influence insomnia. Further studies involving multiple medical universities are needed to investigate the problem in larger populations.

## Figures and Tables

**Table 1:** Characteristics of the study group.

Variables		Study Group (*n* = 308)			
		Mean	Min	Max	SD
Age (years)		20.0	18.0	25.0	1.2
Weight (kg)		67.2	43.0	110.0	12.9
Hight (cm)		1.72	1.53	1.98	0.09
BMI (kg/m_2_)		22.5	16.4	35.4	3.1
AIS–total score		6.7	1.0	18.0	3.3
IPAQ: Vigorous PA		919	0	4800	941
IPAQ: Moderate PA		379	0	2520	426
IPAQ: Low PA		541	0	2772	524
IPAQ: Total PA		1839	0	6213	1161
		*n*		%	
Gender	Female	175		56.8	
	Male	133		43.2	
Economic status	Bad	6		1.9	
	Average	64		20.8	
	Good	185		60.1	
	Very good	53		17.2	
Place of residence	Village	88		28.6	
	A city of up to 100,000	75		24.4	
	The city above 100,000	145		47.1	
Smoking	No	281		91.2	
	Yes	27		8.8	
Coffee consumption	Yes	308		100	
Consumption of energy drinks	I do not drink	278		90.3	
	Several times a week	23		7.5	
	Several times a month	7		2.3	
Alcohol consumption	No	36		11.7	
	Occasionally	213		69.2	
	Once a week	57		18.5	
	Every day	2		0.7	
Stressful situations	Very rarely	20		6.5	
	Several times a month	58		18.8	
	Several times a week	139		45.1	
	Every day	91		29.6	
IPAQ-category	Low	59		19.2	
	Moderate	176		57.1	
	High	73		23.7	
AIS	non-insomnia	194		63.2	
	insomnia	113		36.8	

*n*—number of participants; Min—minimum; Max—maximum; SD—standard deviation; PA—physical activity; AIS—Athens Insomnia Scale.

**Table 2:** Level of physical activity according to gender.

		Low PA	Moderate PA	Vigorous PA	*p*-Value (Chi-Square Test)
Gender	Female	*n* = 46	*n* = 103	*n* = 26	<0.001
		77.97%	58.52%	35.62%	
	Male	*n* = 13	*n* = 73	*n* = 47	
		22.03%	41.48%	64.38%	

**Table 3:** Relationships between insomnia (AIS) and physical activity level (International Physical Activity Questionnaire, IPAQ), (*n* = 308).

	Type of Activity	AIS–Total Score	
		r	P
IPAQ	Vigorous PA	−0.03	0.57
	Moderate PA	−0.05	0.39
	Low PA	0.03	0.63
	Total PA	−0.03	0.57

**Table 4:** Single-factor and multi-factor linear regression analysis assessing the effect of selected variables on insomnia (AIS).

		AIS–Total Score									
		One-Factor Linear Regression Analysis					Multifactorial Linear Regression Analysis				
		B	SE	t	*p*-Value	ß	B	SE	t	*p*-Value	ß
Age (years)		−0.31	0.15	−2.00	0.047	−0.11	-	-	-	-	-
BMI (kg/m_2_)		−0.04	0.06	−0.67	0.498	−0.04	-	-	-	-	-
Vigorous PA		0.00	0.00	−0.57	0.570	−0.03	-	-	-	-	-
Moderate PA		0.00	0.00	−0.87	0.386	−0.05	-	-	-	-	-
Low PA		0.00	0.00	0.48	0.633	0.03	-	-	-	-	-
Total PA		0.00	0.00	−0.56	0.573	−0.03	-	-	-	-	-
Gender	Male	Ref.					-				
	Female	0.59	0.19	3.09	0.002	0.17	-	-	-	-	-
Economic status	Average	Ref.					-				
	Bad	2.25	1.01	2.22	0.027	0.30	-	-	-	-	-
	Good	−0.47	0.41	−1.16	0.249	−0.11	-	-	-	-	-
	Very good	−1.94	0.49	−3.98	<0.001	−0.36	-	-	-	-	-
Place of residence	Village	Ref.					-				
	A city of up to 100,000	0.22	0.30	0.74	0.457	0.05	-	-	-	-	-
	The city above 100,000	−0.06	0.26	−0.22	0.829	−0.01	-	-	-	-	-
Smoking	No	Ref.					-				
	Yes	0.89	0.33	2.68	0.008	0.15	1.23	0.30	4.05	<0.001	0.21
Consumption of energy drinks	No	Ref.					-				
	Several times a week	−0.06	0.63	−0.09	0.927	−0.01	−0.66	0.57	−1.17	0.241	−0.11
	Several times a month	1.25	0.87	1.43	0.154	0.15	1.78	0.78	2.27	0.024	0.21
Alcohol consumption	No	Ref.					-				
	Occasionally	0.23	0.64	0.36	0.719	0.05	-	-	-	-	-
	Once a week	−0.26	0.69	−0.37	0.709	−0.04	-	-	-	-	-
	Every day	0.82	1.78	0.46	0.646	0.08	-	-	-	-	-
Stressful situations	Very rarely	Ref.					-				
	Several times a month	−1.06	0.36	−2.94	0.004	−0.16	−1.06	0.35	−3.03	0.003	−0.16
	Several times a week	0.02	0.29	0.08	0.934	0.00	0.06	0.28	0.23	0.817	0.01
	Every day	2.41	0.32	7.55	<0.001	0.40	2.62	0.32	8.31	<0.001	0.44
Physically active in subjective judgment	No			Ref.							
	Yes	−0.62	0.22	−2.82	0.005	−0.16					
IPAQ-category	Low	Ref.					-				
	Moderate	−0.33	0.26	−1.27	0.20	−0.08	-	-	-	-	-
	High	−0.09	0.31	−0.30	0.76	−0.02	-	-	-	-	-

B—non-standardized regression coefficient B; SE—standard error; t: B/standard error; ß—standardized regression coefficient ß.
